# Adaptive time scales in recurrent neural networks

**DOI:** 10.1038/s41598-020-68169-x

**Published:** 2020-07-09

**Authors:** Silvan C. Quax, Michele D’Asaro, Marcel A. J. van Gerven

**Affiliations:** Donders Institute for Brain, Cognition and Behaviour, Radboud University, Nijmegen, The Netherlands

**Keywords:** Biophysical models, Network models

## Abstract

Recent experiments have revealed a hierarchy of time scales in the visual cortex, where different stages of the visual system process information at different time scales. Recurrent neural networks are ideal models to gain insight in how information is processed by such a hierarchy of time scales and have become widely used to model temporal dynamics both in machine learning and computational neuroscience. However, in the derivation of such models as discrete time approximations of the firing rate of a population of neurons, the time constants of the neuronal process are generally ignored. Learning these time constants could inform us about the time scales underlying temporal processes in the brain and enhance the expressive capacity of the network. To investigate the potential of adaptive time constants, we compare the standard approximations to a more lenient one that accounts for the time scales at which processes unfold. We show that such a model performs better on predicting simulated neural data and allows recovery of the time scales at which the underlying processes unfold. A hierarchy of time scales emerges when adapting to data with multiple underlying time scales, underscoring the importance of such a hierarchy in processing complex temporal information.

## Introduction

Recurrent neural network (RNN) models have become widely used in computational neuroscience to model the dynamics of neural populations as well as in machine learning applications to model data with temporal dependencies. The different variants of RNNs commonly used in these scientific fields can be derived as discrete time approximations of the instantaneous firing rate of a population of neurons^1^. Since such models can mimic the dynamic properties of real neural populations, they are ideally suited to explain neuronal population data such as extracellular multi-unit activity (MUA), functional magnetic resonance imaging (fMRI) or magnetoencephalography (MEG) data. Similarly, it has turned out that these biologically inspired networks enable machines to perform tasks depending on sequential data^2,3^. The interplay between both fields has led to recent advances, enabling such RNNs to solve a wide variety of cognitive tasks^4–6^.

While RNNs have been successfully applied in both computational neuroscience and machine learning, further improvements could be possible through more biologically inspired RNNs. A common assumption that the popular discrete RNN definitions make, when derived from their continuous counterparts, is that of ignoring certain time scales at which the population activity unfolds. The time scale at which processes unfold is an important aspect of modelling dynamic neuronal activity. Some neural responses, like retinal responses to a flashing head light, act at very short time scales, while others, like maintaining the concept of a car in mind, can take very long. Understanding these time scales can give us valuable insights about the nature of the underlying processes and the kind of information that is processed by a neuronal population.

An interesting example of this is the hierarchy of time scales found in the brain^7–10^. Much like the spatial hierarchies found in the visual cortex that ensure an increase in receptive field size along the visual pathway^11^, there is evidence for a hierarchy of time scales with lower visual areas responding at shorter time scales and higher visual areas at longer time scales^12^. At the neural level, cell assemblies of strongly interconnected neurons that serve as representations of static or dynamic events of different duration have been suggested to develop a hierarchical temporal structure^13,14^.

A tempting explanation for the emergence of such a hierarchy is the hierarchical causal structure of the outside world that shapes the representations of the brain^8,15–17^. For example, a car passing by leads to changes in neuronal activity on a short time scale in the retina, where the amount of light reaching a receptor could suddenly change, while on the other hand, neurons in V4 or IT, encoding the concept ‘car’ would change activity at a much longer time scale. As for the spatial receptive field, one can define a property of the neurons (or of the coding unit more in general, be it an artificial neuron or a population of neurons in a voxel) that defines the region of time of interest for a stimulus to trigger its activity. The idea of such temporal receptive windows of a neuron that represents the length of time before a response during which sensory information may affect that response has been suggested^7^, emphasizing the important role of time scales in the neural responses of different cortical areas.

While a hierarchy of time scales can thus be important for processing temporal information, there is no specific time scale parameter in the RNNs commonly used for modelling. Often either one or multiple implicit assumptions about the time scales at which the networks operate are made in the definition of the RNNs used. In computational neuroscience models it is often assumed that either the firing rate closely tracks the intrinsic current, or the intrinsic current closely tracks the firing rates. Models used for artificial intelligence problems on the other hand, tend to ignore time scales both for the intrinsic currents and firing rates (see “Methods” for further details).

By allowing our models to learn the time scales at which processes operate, a closer relation to the hierarchies of time scales of our natural world could be achieved, allowing a model to perform better. At the same time this would help in interpreting the role different neurons of the model perform by linking their time scales to the underlying processes.

Here we investigate whether a more biologically plausible model with learnable intrinsic time parameters is able to recover the time scales of the processes underlying a given dataset and whether the flexibility of learning these time scales is beneficial for the performance of the model. We find an improvement in performance compared to the commonly used approximations for RNN models and an increase in the memory capacity of the RNN models. At the same time we can recover the time scales of the underlying processes from the data, opening interesting opportunities to improve our understanding of hierarchies of time scales in the brain.

## Methods

### Synaptic coupling between neurons

Deriving the equations governing the communication between neurons requires specification of a mechanism for synaptic transmission, referring to how incoming spikes generate synaptic currents through the release of neurotransmitters in the synaptic cleft^1^. That is, we need to make explicit how the synaptic current *I* arises. Consider a presynaptic neuron indexed by *k* whose spike times are given by $\{ t^k_1,\ldots,t^k_N \}$, with N the number of spikes. Formally, this spike train is fully described by the neural response function

1 $$\begin{aligned} \rho _k(\tau ) = \sum _{i} \delta \left( \tau -t^k_i \right), \end{aligned}$$

where the idealized action potentials are given by Dirac $\delta$ functions. The total synaptic current received by a neuron *n* is modeled as

2 $$\begin{aligned} I_n(t) = \sum _k w_{nk} \sum _{t_i < t} K(t - t^k_i) = \sum _k w_{nk} \int _{-\infty }^t d \tau K(t - \tau ) \rho _k(\tau ), \end{aligned}$$

where $w_{nk}$ is a measure of the efficacy of the synapse from neuron *k* to neuron *n* and $K(\cdot )$ determines how presynaptic spikes are transformed into synaptic currents.

A common choice for *K* is simply $K(s) = q \delta (s)$, where *q* is the charge injected via a synapse with strength $w_{nk} = 1$. A more realistic choice for *K* is to assume that the synaptic current has a finite duration, as modeled by the exponential kernel

3 $$\begin{aligned} K(s) = \frac{q}{\tau _s} \exp \left( - \frac{s}{\tau _s} \right), \end{aligned}$$

where $\tau _s$ is the time constant. A detailed description of why this kernel function is a suitable approximation can be found in^18^.

### From spiking to rate-based models

An alternative to spiking neuron models is to assume that neural coding is driven by the rate at which neurons fire, rather than the exact arrival times of individual spikes. A mean firing rate over a temporal window of length *T* can simply be computed from individual spike times as

4 $$\begin{aligned} \nu = \frac{1}{T} \int _0^T d \tau \rho (\tau ) \,. \end{aligned}$$

The use of mean firing rates to quantity neural responses dates back to the work of Adrian^19^, who showed that the firing rate of muscular stretch receptors is related to the force applied to the muscle. However, a problem associated with the use of mean firing rates is that it severely limits the speed at which information can be processed given the requirement to integrate over time. This is unrealistic given the very rapid response times observed in visual detection tasks^20^.

An alternative formulation is provided by assuming that the rate code captures a population average by counting the number of spikes produced by a population consisting of *M* neurons over a short time interval $\Delta t$. That is, we interpret the firing rate as a population activity, given by^21^:

5 $$\begin{aligned} r(t) = \frac{1}{\Delta t} \frac{1}{M} \int _t^{t+\Delta t}d \tau \sum _{m=1}^M \sum _{i} \delta \left( \tau - t^m_i\right) \,. \end{aligned}$$

Under this interpretation, we view artificial neurons as models of neuronal populations that are influenced by presynaptic populations in a homogeneous manner and collectively produce a firing rate as output. The population activity may vary rapidly and can reflect changes in the stimulus conditions nearly instantaneously^22^.

To compute the postsynaptic current induced by the presynaptic firing rates, we replace the neural response function in Eq. (2) by the firing rate, to obtain

6 $$\begin{aligned} I_n(t) = \sum _k w_{nk} \int _{-\infty }^t d \tau K(t - \tau ) r_k(\tau ) \,. \end{aligned}$$

Using an exponential kernel (3) with $q=1$ and taking the time-derivative of Eq. (6) yields

7 $$\begin{aligned} \begin{aligned} \frac{d I_n}{d t}&=-\frac{1}{\tau _s}\sum _k w_{nk} \int _{-\infty }^t d \tau \frac{e^{-\frac{(t - \tau )}{\tau _s}}}{\tau _s} r_k(\tau ) \ +\ \frac{1}{\tau _s}\sum _k w_{nk} r_k(t)~, \end{aligned} \end{aligned}$$

where the Leibniz rule is applied to calculate the derivative of the integral (see Appendix S1 for a derivation). Substituting the first term on the right hand side for Eq. (6) and using a compact vector notation this simplifies to

8 $$\begin{aligned} \tau _s \frac{d I_n}{d t} = -I_n + {{\mathbf {w}}}_n^\top {{\mathbf {r}}}, \end{aligned}$$

with ${{\mathbf {w}}}_n = (w_{n1},\ldots ,w_{nK})^\top$ and ${{\mathbf {r}}} = (r_{1},\ldots ,r_{K})^\top$. The kernel time constant, $\tau _s$, thus determines the time scale of the differential process.

To complete the model, we assume that the synaptic input $I_n$ directly influences the firing rate $r_n$ of a neuron. Due to the membrane capacitance and resistance the firing rate does not follow the current instantaneously, but with a delay determined by a time constant $\tau _r$. That is,

9 $$\begin{aligned} \tau _r \frac{d r_n}{d t} = - r_n + f(I_n)\ , \end{aligned}$$

where *f* is an activation function (a static nonlinearity) which translates currents into firing rates. Equations (8) and (9) together define the firing-rate model.

### Discrete approximation of the recurrent dynamics

As mentioned before, firing-rate models provide a biological counterpart for recurrent neural networks, where RNN units reflect the average activity of a population of neurons. The equations for the standard RNN follow from the continuous equations through discretization.

We use the forward Euler method to numerically approximate the solution to the differential equations (8) and (9) and further generalize by assuming that at each point in time, the firing rates are influenced by sensory inputs ${{\mathbf {x}}}$. We obtain

10 $$\begin{aligned} {{\mathbf {I}}}_{t}=  {} {{\mathbf {I}}}_{t-1} + \tau _s^{-1}(- {{\mathbf {I}}}_{t-1} + {{\mathbf {W}}} {{\mathbf {r}}}_{t-1} + {{\mathbf {U}}}{{\mathbf {x}}}_t ) \Delta t \end{aligned}$$

11 $$\begin{aligned} {{\mathbf {r}}}_{t}=  {} {{\mathbf {r}}}_{t-1} + \tau _r^{-1} (- {{\mathbf {r}}}_{t-1} + {{\mathbf {f}}}\left( {{\mathbf {I}}}_{t}\right) ) \Delta t, \end{aligned}$$

where ${{\mathbf {W}}}$ and ${{\mathbf {U}}}$ are matrices whose values represent synaptic weights, $\Delta t$ indicates discrete time steps. We can rewrite the last equations, defining $\alpha _s = \Delta t / \tau _s$ and $\alpha _r = \Delta t / \tau _r$, as (In what follows, we will refer to $\alpha _r$, $\alpha _s$ as ‘rate constants’, keeping in mind their relation to $\tau _s$ and $\tau _r$. Rate constants expressed as $\alpha$ are just more practical for our purpose.):

12 $$\begin{aligned} {{\mathbf {I}}}_{t}=  (1 - \alpha _s) {{\mathbf {I}}}_{t-1} + \alpha _s \left( {{\mathbf {W}}} {{\mathbf {r}}}_{t-1} + {{\mathbf {U}}}{{\mathbf {x}}}_t \right) \end{aligned}$$

13 $$\begin{aligned} {{\mathbf {r}}}_{t}=  (1 - \alpha _r) {{\mathbf {r}}}_{t-1} + \alpha _r {{\mathbf {f}}}\left( {{\mathbf {I}}}_{t} \right) \ . \end{aligned}$$

These are going to be the key equations of the model under investigation.

### Time scale assumptions

In literature, several variants of Eqs. (12) and (13) are commonly used to model recurrent dynamics. These variants have implicitly different underlying time scale approximations. Here we review the two approximations that are at the basis of commonly used rate-based RNN models in computational neuroscience and artificial intelligence (AI), derived from our set of equations above. Typically, either the process of integrating the presynaptic current, or the process of generating postsynaptic rate activity, are modeled in literature as instantaneous; a choice which resides in the assumption that one of the two processes is much faster than the other^1^. We will refer to these complexity reduction choices as *extreme cases* for the time constants. Despite the popularity of such choices, it is often neglected in literature why this approximation is made. Furthermore, the implicit time constants used in these models are typically not motivated.

The first approximation assumes that the time constant for the firing rate $\tau _r$ is much larger than that of the current $\tau _s$. In this case, the current closely tracks the firing rates and we can assume that, for the *n*th-neuron, $I_n(t) = {{\mathbf {w}}}_n^\top {{\mathbf {r(t)}}}$. By using this substitution, generalizing to multiple neurons and adding the external input again, the equations describing the dynamics at the network level are then given by

14 $$\begin{aligned} {{\mathbf {I}}}_{t}=  {{\mathbf {W}}} {{\mathbf {r}}}_{t-1} + {{\mathbf {U}}}{{\mathbf {x}}}_{t-1} \end{aligned}$$

15 $$\begin{aligned} {{\mathbf {r}}}_{t}=  {{\mathbf {r}}}_{t-1} + \alpha _r \left( - {{\mathbf {r}}}_{t-1} + {{\mathbf {f}}}\left( I_{t} \right) \right), \end{aligned}$$

with ${{\mathbf {W}}} = \left[ {{\mathbf {w}}}_1, \ldots , {{\mathbf {w}}}_K\right] ^\top$ and ${{\mathbf {f}}}$ a vector-valued activation function.

The second approximation on the other hand, assumes that the time constant for the firing rate $\tau _r$ is much smaller then that of the current $\tau _s$. The firing rate then closely tracks the current and we can assume $r_n = f(I_n)$. The model is then fully described by Eq. (8). Generalizing to multiple neurons, we obtain

16 $$\begin{aligned} {{\mathbf {I}}}_{t}=  {{\mathbf {I}}}_{t-1} + \alpha _s \left( - {{\mathbf {I}}}_{t} + {{\mathbf {W}}} {{\mathbf {r}}}_{t-1} + {{\mathbf {U}}}{{\mathbf {x}}}_t\right) ) \end{aligned}$$

17 $$\begin{aligned} {{\mathbf {r}}}_{t}=  {{\mathbf {f}}}\left( {{\mathbf {I}}}_{t} \right) \ . \end{aligned}$$

With respect to our discrete-time Eqs. (12) and (13), these two extreme cases correspond respectively to the choices for the rate constants, $\alpha _s = 1$ (Eqs. (14) and (15)) and $\alpha _r = 1$ (Eqs. (16) and (17)). Studies in computational neuroscience that implement RNNs as modeling tool often use the extreme case of Eqs. (16) and (17)^23–25^.

Studies in AI, on the other hand, tend to ignore both processes, equivalent to assuming both $\tau _r = \Delta t$ and $\tau _s = \Delta t$. These models, referred to as Elman networks^26^, are determined by

18 $$\begin{aligned} {{\mathbf {I}}}_{t}=   {{\mathbf {W}}} {{\mathbf {r}}}_{t-1} + {{\mathbf {U}}}{{\mathbf {x}}}_{t} \end{aligned}$$

19 $$\begin{aligned} {{\mathbf {r}}}_{t}= {{\mathbf {f}}}\left( {{\mathbf {I}}}_{t} \right) \ . \end{aligned}$$

Setting the rate constants $\alpha _r = 1$ and $\alpha _s = 1$, as in Eqs. (18) and (19), means that the synaptic current and the firing rate both follow instantaneously the presynaptic firing rates. Thus, every neuron acts as a non-linear filter that carries no memory of its previous states internally. The activity of a neuron is influenced by the recent firing rate history only through the recurrent weights.

Since rate constants ensure an easier notation for the network equations that will follow we will from now on express everything in terms of the rate constants $\alpha _r$ and $\alpha _s$, keeping in mind their direct relation with their respective time constant.

### Optimizing rate constants

While deliberately disregarding one or both of the dynamical processes simplifies the equations describing an RNN, such a simplification could prevent us from achieving optimal performance and gaining valuable insight into the dynamics of the underlying process. We aim here to show that, from a functional perspective such enrichment of the internal dynamics increases the performance of RNNs commonly used in the literature. At the same time it could provide us with valuable insight into the dynamics of the underlying data that the RNN tries to explain.

Previous works have experimented with manually setting the rate constants of the dynamic process to enhance the expressiveness of RNNs^25,27^. While it is possible to come up with rate constant values deemed biologically relevant, a much more interesting approach would be to optimize the rate constants to adapt to the process at hand. The idea that rate constants can be inferred from the data and not set a priori, with the aim to better describe the observed data, has been suggested previously in the continuous time regime^28^. Other work has optimized networks with a single rate constant in the discrete approximation regime through numerical integration^29^, though limited by the computationally intensive integration process.

Here, instead, we optimize the rate constants of the RNN using the backpropagation-through-time (BPTT) algorithm, alongside the other parameters of the network.

### Adaptive time scales recurrent neural network

**Figure 1:**
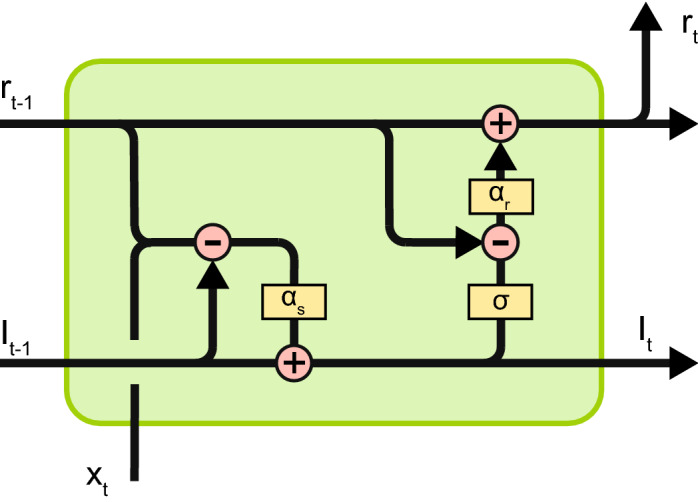
Diagram of the ARU implementation with internal state and rate constants as described by Eqs. (12) and (13). Pink circles represent pointwise operators (plus and minus). Yellow rectangles represent layers (scaling layer, $\alpha _r$ and $\alpha _s$, or a sigmoid layer, $\sigma$). Combining arrows represent a concatenation operation and splitting arrows represent a copying operation.

To investigate the potentially beneficial role rate constants can play in the performance and interpretability of RNNs, we developed an RNN model which can adapt the rate constants at which dynamic processes unfold. The model was developed using the Chainer package for automatic differentiation^30^. The model consisted of an RNN with hidden units that generate a firing rate according to Eqs. (12) and (13), referred to as adaptive recurrent units (ARUs) throughout the next sections (Fig. 1). The output units transform the hidden units activity in a readout layer. All layers were fully connected. The non-linear transformation of the synaptic currents into firing rate is modelled by a sigmoid function, unless otherwise specified.

The complete model is then given by

20 $$\begin{aligned} {{\mathbf {I}}}_{t} &=  (1 - \alpha _s) {{\mathbf {I}}}_{t-1} + \alpha _s \left( {{\mathbf {W}}} {{\mathbf {r}}}_{t-1} + {{\mathbf {U}}}{{\mathbf {x}}}_t\right) )\nonumber \\ {{\mathbf {r}}}_{t} &=  (1 - \alpha _r){{\mathbf {r}}}_{t-1} + \alpha _r \left( {{\mathbf {f}}}\left( {{\mathbf {I}}}_{t} \right) \right) \nonumber \\ {{\mathbf {y}}}_{t} &=  {{\mathbf {f}}}\left( {{\mathbf {V}}}{{\mathbf {r}}}_t \right), \end{aligned}$$

where ${{\mathbf {x}}}$ is the input signal, ${{\mathbf {y}}}$ is the output, ${{\mathbf {W}}}, {{\mathbf {U}}}, {{\mathbf {V}}}$ weight matrices, ${{\mathbf {I}}},{{\mathbf {r}}}$ are the *N*-dimensional state variables referring to the postsynaptic current and the firing rates, with *N* the number of hidden (recurrent) units, and ${{\mathbf {f}}}$ a sigmoid function. The initial states of the synaptic current and firing rate were also learned through the BPTT algorithm^31^. We always used an additional input with a fixed value of one as a bias term.

### Simulations

To investigate whether the explicit modelling of time scales benefits performance and leads to recovery of the relevant time scales of the dynamics of the data, several simulations were performed. Artificial training data was created that has temporal dynamics reflective of a certain time scale by using our previously described model with a certain set of rate constants as a generative model to produce data. A random input pattern that consisted of a two-valued signal was generated by drawing samples from a white noise distribution between 0 and 1, and applying a smoothing Savitzky-Golay filter^32^.

The data consisted of a series of 500 samples (400 for training and 100 for validation sets) of input-output pairs unfolding over 20 time steps. Target networks are structured as explained in Fig. 1, with two units in the input layers, 10 units in the hidden layer and two units in the output layer (unless stated otherwise). The data generated this way reflects the time scales of the underlying generating process, thus expressing slower dynamics for $\alpha _s$, $\alpha _r$ close to one and expressing fast dynamics for $\alpha _s$, $\alpha _r$ close to zero.

In the simulations, this data was used to optimize networks to learn to produce the output data from the input data. The mean-squared-error between the outputs of the network and the training data set was minimized via BPTT. During training, the data was divided into minibatches. For optimization, the Adam^33^ optimizer was used with a learning rate of 0.001.

#### Influence of rate constants

We first ran a series of grid search experiments over the $\alpha _s$, $\alpha _r$ parameter space, where the rate constants of the trained network were fixed to a certain value, in order to visualize the loss landscape and check the relevance of the rate constants for performance. To avoid confusion, the rate constants of the trained network will from here on be indicated as ${\hat{\alpha }}_s, {\hat{\alpha }}_r$. During the grid search the ${\hat{\alpha }}_s, {\hat{\alpha }}_r$ values tested ranged from 0.001 to 1.3 to cover a biologically plausible range (Values for ${\hat{\alpha }}_s, {\hat{\alpha }}_r$ higher than one indicate a negative correlation between subsequent time steps, which is not biologically plausible but were included out of exploratory interest.). For each pair of rate constants $({\hat{\alpha }}_s,{\hat{\alpha }}_r)$ a network was trained over the generated data, minimizing the mean squared error between the outputs of the network and the training data set. Six different combinations of rate constants were used to generate data, that is ($\alpha _s,\alpha _r) \in \{ (0.34,0.68),(0.34,1.0),(0.68,0.34),(0.68,0.68),(0.89,0.89),(0.14,0.14)\}$.

#### Learning optimal rate constants

Next, we investigated the idea of learning the best rate constants by BPTT and check whether the rate constants are recovered correctly and lead to an improvement in performance. For two combinations of rate constants ($\alpha _s=0.34$, $\alpha _r=0.68$ and $\alpha _s=0.68$, $\alpha _r=0.34$) we trained 20 repetitions of networks with optimizable rate constants. The performance was compared with the standard Elman network with fixed rate constants of ${\hat{\alpha }}_s=1$, ${\hat{\alpha }}_r=1$.

To investigate the influence of network size on the ability to recover the rate constants, networks with different numbers of hidden units were trained (5, 10, 30 and 100 units). A grid search over fixed rate constants in the range 0.001 to 1.3 was performed to identify the loss landscape. On top of that, networks with learnable rate constants were trained, with 20 repetitions per network size to investigate the distribution of learned rate constants.

The influence of the activation function was compared by performing simulations with networks using the sigmoid and rectified linear unit activation functions.

#### Learning individual rate constants

In the previous simulations we only used models with rate constants that were shared across all units in the network. To investigate whether having individual rate constants per unit can help in learning processes with a range of underlying time scales, data was generated with three different distributions of rate constants. Rate constants were drawn from a Gaussian distribution truncated between 0 and 1. The mean for all three distributions was 0.5, the standard deviation varied from 0.1 to 0.3. The networks were extended with individual rate constants per unit and 20 repetitions were trained per data set. The standard deviation of the learned rate constants was compared with the standard deviation of the rate constants used to generate the data, to infer whether the original distribution was recovered. To investigate whether there was also a benefit in performance the network with individual rate constants was compared with an Elman network and a network with global rate constants.

#### Testing memory capacity

The memory capacity of the resulting networks was investigated by generating white noise data where the network had to remember the input *N* steps back, with $N \in \{5,10,20,30,40\}$. To generate a data set with a common underlying data distribution, one data set was generated by low-pass filtering a white noise process to 20 Hz. For every memory length sequences are generated by iterating through windows of 250 ms of data using time-step $\Delta t = 100/\text{N} \,  \text{ms}$. The Elman network, the network with global rate constants and the network with individual rate constants were all trained for 20 repetitions on every memory length task.

## Results

### Optimal rate constants improve performance

To investigate whether explicitly modelling rate constants in an RNN benefits performance compared to the commonly used approximations (see “Methods”), we performed several simulations. Artificial data with several combinations of rate constants, $(\alpha _s,\alpha _r)$, was created by using an RNN as generative model (see “Methods” for further details). A grid search for parameters (${\hat{\alpha }}_s,{\hat{\alpha }}_r$) over the range 0.001 to 1.3 was performed to identify the combination of rate constants resulting in the best performance. In these simulations, we were interested in seeing whether a network trained with rate constants matching those of the generating process improves performance. Alternatively, there could be a general optimal choice for ${\hat{\alpha }}_s$, ${\hat{\alpha }}_r$ that is independent of the time scale of the generated data, or the rate constants could be completely irrelevant and compensated by the complex recurrent dynamics of the network.

**Figure 2:**
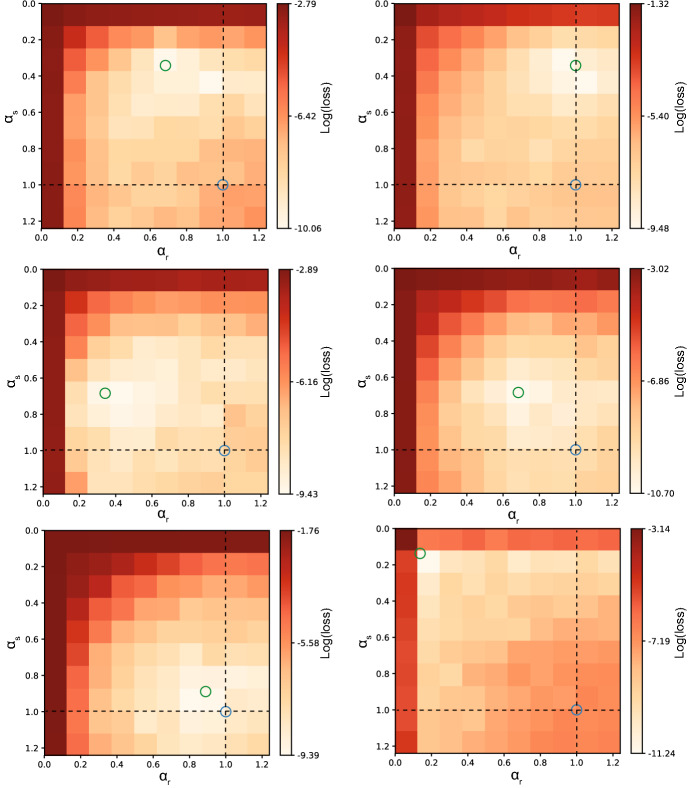
Grid search reveals optimal rate constants improve performance. Data were generated for six different combinations of target rate constants. A grid search was performed for each of these target pairs, as shown in the different panels (the target rate constants are marked by the green circle in every panel). The dashed lines indicate the approximation where either ${\hat{\alpha }}_s= 1$ or ${\hat{\alpha }}_r= 1$. The intersection of both dashed lines indicates the Elman solution (blue circle). The region of lower loss for all generated data examples centers around the actual values ($\alpha _s, \alpha _r$) used to generate the data. This indicates that there is a performance benefit from choosing the correct rate constants that is not compensated for by the recurrent interactions between neurons.

The lowest loss is found for values of rate constants around the values that were used to generate the data (Fig. 2). This region could differ strongly from the commonly used approximation where either one rate constant is ignored (dashed lines) or both rate constants are ignored (blue circle). The shape of the loss landscapes changed according to the values of the target rate constants, thus indicating that there is not a single choice of rate constants that is optimal for any kind of data, irrespective of the underlying generative process.

These results show that the network can recover the rate constants of the underlying process that generated certain data. Despite the rich dynamics that RNNs can develop, this is not able to fully compensate for a choice of rate constants that is different from the optimal one. At the same time we do not find a symmetric solution (symmetric in the sense that exchanging ${\hat{\alpha }}_s$ and ${\hat{\alpha }}_r$ results in the same performance after training), which means that ${\hat{\alpha }}_r$ and ${\hat{\alpha }}_s$ themselves cannot compensate for each other. Both rate constants are thus important and an approximation with a single rate constant will lead to sub-optimal solutions.

### Learning optimal rate constants through backpropagation

From the previous results it becomes clear that choosing the right combination of rate constants is beneficial to the performance of an RNN. Most of the time we do not know the underlying time scales of our data though. For this reason it could be useful to let the model learn the rate constants via BPTT (We investigated whether these parameters can be inferred with gradient descent algorithms. Similar results hold for Stochastic Gradient Descent and Adam optimizer^33^.).

**Figure 3:**
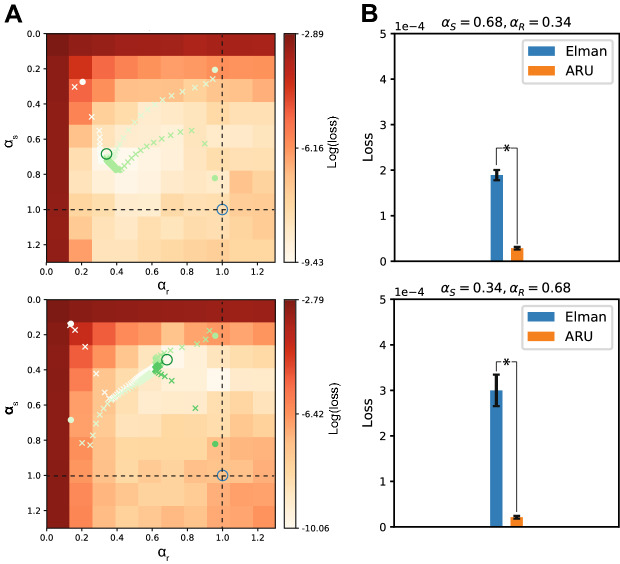
Optimizing rate constants through backpropagation. Instead of choosing the rate constants manually, the BPTT algorithm was used to optimize them, along with the rest of the parameters of the network. (**A**) For two combinations of target rate constants ($\alpha _s=0.684$, $\alpha _r=0.34$, top panel) and ($\alpha _s=0.34$, $\alpha _r=0.68$, bottom panel) the optimization of the rate constants is plotted over the grid search results of Fig. 2. The target rate constants are marked by a green circle. Different initializations of the rate constants were tested, as indicated by the filled circles with different shades of green. The learned values after each epoch are marked by a cross of the same color. The dashed lines indicate the approximation where either ${\hat{\alpha }}_s= 1$ or ${\hat{\alpha }}_r= 1$. The intersection of both dashed lines indicates the Elman solution (blue circle). Independent of initialization, the learned rate constants all converge to the target rate constants, closely recovering the correct values. (**B**) The performance of the ARU model with learnable rate constants was compared with the classical Elman model. The ARU model performed significantly better than the Elman network (*$p < 1 \cdot 10^{-11}$ and *$p < 1 \cdot 10^{-6}$ for the top and bottom panels, respectively) for both data sets (error bars represent standard error over 20 repetitions).

The learning trajectories of the rate constants for two example choices of rate constants are shown in Fig. 3A, where the learning trajectories of (${\hat{\alpha }}_s, {\hat{\alpha }}_r$) is plotted upon the loss landscape of the same simulations as in Fig. 2 (top and middle left panels, i.e. ($\alpha _s=0.34$, $\alpha _r=0.68$) and ($\alpha _s=0.68$, $\alpha _r=0.34$). Independent of the initialization of the rate constants, they clearly converge towards the region of lowest loss obtained from the grid search experiment and recovers the the rate constants used in the generative process. Thus the rate constants are identifiable and can be learned effectively through BPTT. This means that we can infer information about the time scale of the data by training models to predict dynamic responses from a set of input stimuli. At the same time, the adaptive rate constants significantly improved performance over the standard Elman units (Fig. 3B), showing the importance of these rate constants.

### Influence of network size on time scale recovery

**Figure 4:**
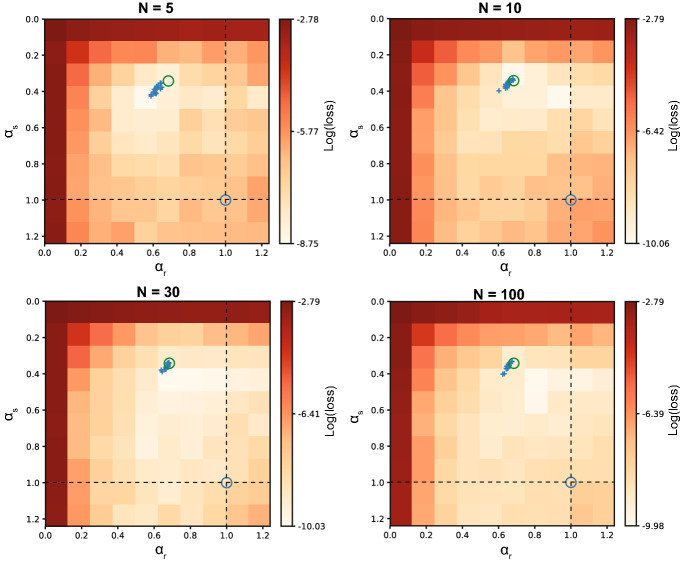
Effect of network size on recovering time parameters. The number of hidden units in the trained model *N* was varied to test the effect on the loss landscape. The target model that generated the data of these simulations was equipped with rate constants ($\alpha _s=0.34$, $\alpha _r=0.68$) as indicated with the green circle, and 10 hidden units. Networks with 5, 10, 30 and 100 hidden units were trained on the generated data. Both a grid search over fixed rate constants was performed and networks with adaptive rate constants were trained with their final learned rate constants indicated as blue crosses (20 repetitions). The dashed lines indicate the approximation where either ${\hat{\alpha }}_s= 1$ or ${\hat{\alpha }}_r= 1$. The intersection of both dashed lines indicates the Elman solution (blue circle). A larger network size did not decrease the ability of the network to recover the underlying target rate constants.

Simple Elman networks can be seen as universal approximators when given enough hidden units^34^. Therefore, it is possible that a benefit in performance and the recovery of the actual rate constants is less in larger networks. We tested networks with different numbers of hidden units in the trained model, as shown in Fig. 4. From these results it is clear that more hidden units do not make the rate constants less important with respect to model adaptability: the region of lower loss around the optimal point does not expand as the number of hidden units increases. When learning the rate constants, the precision of the recovery does not change with network size either (Fig. 4, blue crosses). Despite the fact that larger networks could learn more complex dynamics, possibly overcoming a non-optimal choice of rate constants, the ability to effectively recover rate constants of the underlying dynamical process remains. This is promising for future applications on more complex dynamical problems requiring large-scale networks, for example for explaining neural responses in the brain.

### Time scale recovery depends on activation function

**Figure 5:**
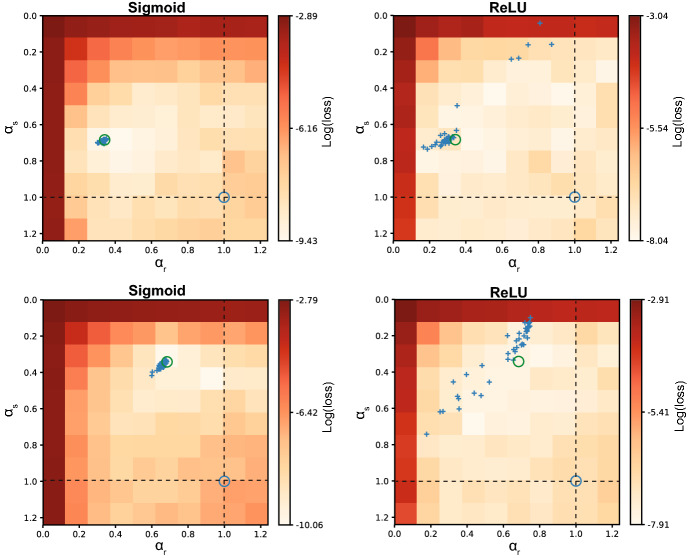
Effect of activation function on recovering time parematers. Networks were either equipped with a sigmoid activation function (left) or a ReLU activation function (right). For two combinations of target rate constants ($\alpha _s=0.68$, $\alpha _r=0.34$, upper panels) and ($\alpha _s=0.34$, $\alpha _r=0.68$, lower panels) a grid search was performed over fixed rate constants and networks with adaptive rate constants were trained with their final learned rate constants indicated as blue crosses (40 repetitions). Target rate constants are indicated with a green circle. The dashed lines indicate the approximation where either ${\hat{\alpha }}_s= 1$ or ${\hat{\alpha }}_r= 1$. The intersection of both dashed lines indicates the Elman solution (blue circle). The networks with a sigmoid activation function have a region of lowest loss contained around the target values. However networks with an ReLU activation function have a much wider basin of rate constants associated with minimal loss. Learned rate constants do not recover the target rate constants for the ReLU activation function uniquely but form a band symmetric around the diagonal $\alpha _s=0$, $\alpha _r=0$. The grid search loss region for the ReLU activation function is also symmetric around the diagonal, indicating an interchangeability of rate constant ${\hat{\alpha }}_s$ and ${\hat{\alpha }}_r$. This could be the result of the interchangeability in the linear regime of the ReLU activation function (see Appendix S2).

A diverse set of activation functions can be used for the nonlinearities in RNNs. Our previous simulations were done with the commonly used sigmoid activation function. Since it is possible that the recovery of the correct rate constants depends on the choice of activation function for the network, we also tested another commonly used activation functions, namely the rectified linear unit (ReLU) (Fig. 5).

While the loss landscape clearly shows a region of lower loss around the rate constants used to generate the data in the case of sigmoid activation functions, the loss landscape becomes more shallow and wider in the case of ReLU activation functions, making it harder to identify a unique combination of rate constants that leads to the lowest loss. The loss region also becomes more symmetric with respect to the diagonal ${\hat{\alpha }}_s = {\hat{\alpha }}_r$. An explanation for this can be that a linear activation function (when in the linear regime) makes the two dynamical processes of each unit (Eqs. 8, 9) reducible to one, in which we have new rate constants which are invariant for exchange of ${\hat{\alpha }}_s, {\hat{\alpha }}_r$ (see Appendix S2 for a derivation). When optimizing the rate constants with BPTT, we also find that there is no good recovery of the original rate constants (Fig. 5, blue crosses). If our goal is thus to recover the rate constants governing the data at hand, the ReLU activation function is not suitable and the sigmoid activation function is a better option.

### Learning a range of time scales

In the previous sections, the learned rate constants were shared over all neurons in the network. While this might be sufficient to describe processes with a single underlying pair of rate constants, it might not be optimal to describe processes that are governed by a whole range of time scales. To enhance the expressiveness of the network, we learn individual rate constants per neuron. Besides increasing performance it could possibly provide insight in the range of time scales underlying the data, where data with a single underlying time scale would lead to narrow range of learnt rate constants, and data with multiple different underlying time scales would lead to a broad range of learned rate constants.

To test whether such a network that can learn individual rate constants per neuron is informative about the range of time scales underlying a process, we generated data with different distributions of rate constants drawn from a truncated Gaussian distribution. Subsequently four different neural network models were trained. A network with global adaptive rate constants and a network with local adaptive rate constants per unit were compared against a standard Elman network and a network composed of the frequently used gated recurrent units (GRU’s)^35^.

**Figure 6:**
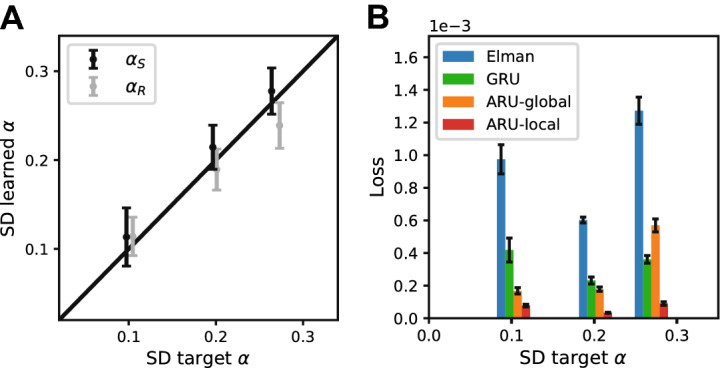
Recovering a distribution of rate constants. Data was generated for three rate constant distributions with increasing standard deviation (SD). Four different network models were tested, a standard Elman network, a GRU network, a network with local adaptive rate constants and a network with global adaptive rate constants. (**A**) Networks trained on this data adopted a range of rate constants with similar SD. (**B**) The model with local rate constants performs best on the data, improving over the model with global rate constants, especially on data generated from a distribution with high SD (error bars represent standard error over 20 repetitions).

The learned distribution of rate constants was compared with the distribution of rate constants used to generate the data. Figure 6A shows the standard deviation of the learned rate constants versus the standard deviation of rate constants used to generate three different datasets. The network clearly learns to adjust its rate constants to the underlying distribution of the data. The range of rate constants is recovered such that it can inform us about the underlying rate constant distribution. At the same time the network with individual rate constants outperforms the network without individual rate constants, the Elman network and the GRU network (Fig. 6B). The largest performance difference is found for the dataset generated with the broadest range of rate constants, demonstrating the benefit of learning individual rate constants on datasets with different underlying processes. While the GRU network has three times as many parameters it performs worse than the network with individual rate constants, showing the importance of a network that is architecturally similar to the process it tries to model, making a case for such biologically plausible networks when modelling neural processes.

### Adaptive time scales increase memory capacity

**Figure 7:**
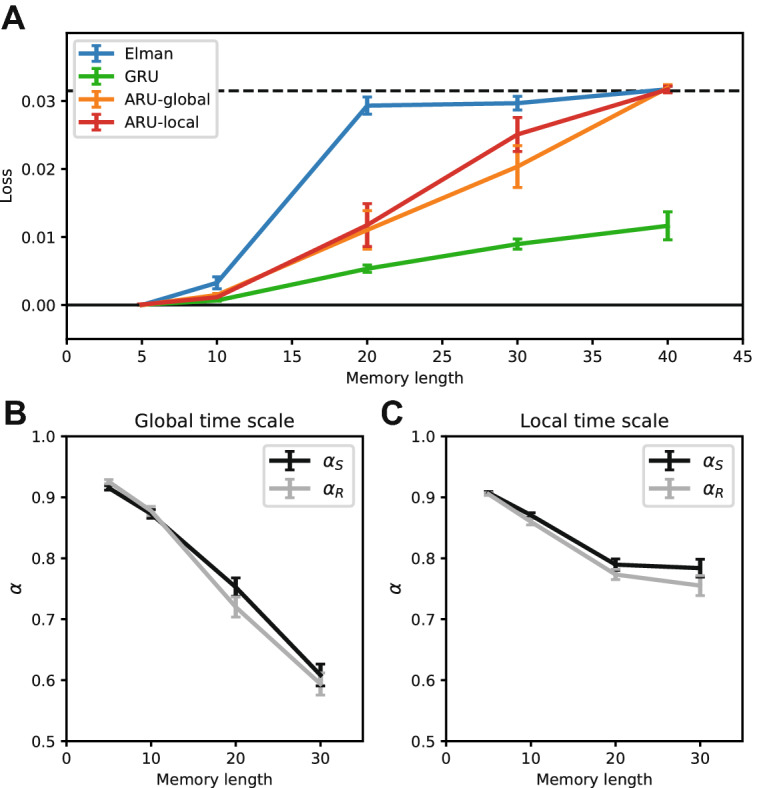
Adaptive time scales increase memory capacity. Three different model variations were tested on a memory capacity task. The number of time steps the input had to be remembered varied from 5 to 40. (**A**) The model using standard Elman units had the lowest memory capacity, performing better than chance up to memory lengths of 10 time steps. Both the model with global and local rate constants performed better than chance up to memory lengths of 30 time steps (dashed line represents chance level, which is defined as predicting the average over all time steps). (**B**, **C**) The models with adaptive rate constants learned slower rate constants with increasing memory lengths, increasing the capacity to maintain memories over longer time scales (error bars represent standard error over 20 repetitions).

Adaptive time scales can lead to slower dynamics in a trained network. This should result in an enhanced capacity to retain memory over longer time scales. To test this idea we designed a simple memory task where the memory length could be varied^36^. In this simple task networks had to remember the input that the network received *N* time steps back, with $N \in \{5,10,20,30,40,50\}$. The longer back in time the network had to remember its input, the more difficult the task became, demanding enhanced memory capacity. We compared the standard Elman network and GRU network, with a network with a pair of global adaptive rate constants, and a network with local adaptive rate constants for every unit. Figure 7A shows the performance of the networks on the different memory lengths. The standard Elman network is no longer able to learn the task for retention periods beyond 10 steps, while both networks with adaptive rate constants learn better than chance level until memory lengths of 40 steps. The memory capacity of the GRU network is even larger, but this network uses three times as many parameters. To investigate how the adaptive rate constants played a role in this increased memory capacity, we averaged the rate constants over successfully trained models for the different memory lengths. The learned rate constants decreases for longer memory lengths for both network models (Fig. 7B,C), indicating that the slower internal dynamics helped maintain memories over longer time windows.

### A hierarchy of time scales

**Figure 8:**
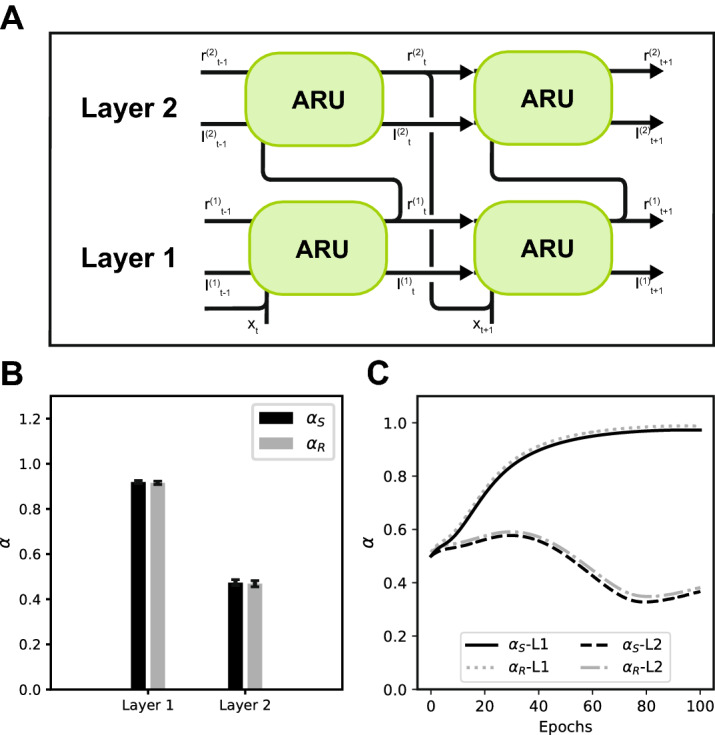
Learning a hierarchy of time scales. (**A**) A two-layer recurrent neural network was created, consisting of ARUs with a global rate constant per layer. Both layers were connected through feedfoward and feedback connectivity. (**B**) A hierarchy of time scales emerged with the rate constants learned by the first supporting much faster dynamics than the rate constants learned by the second layer (error bars represent standard error over 20 repetitions). (**C**) Example of learning trajectory of rate constants in both layers over 100 training epochs (trajectories for $\alpha _r$ have been offset by 0.01 for visibility).

Recent findings have shown a hierarchy of time scales in the visual cortex with lower visual areas responding at faster time scales to changes in visual input and higher visual areas responding at slower time scales^7^. This raises the question whether such a hierarchy of time scales also emerges when we stack multiple layers of ARU units, each able to adapt its own rate constant, on top of each other. To test this, data with a combination of a fast (10 Hz) and slow (2 Hz) sinusoidal signal was generated. A two-layer recurrent network was created consisting of ARU units with feedforward and feedback connections between both layers (Fig. 8A). As a task, the first layer of the network had to predict the next time step of the signal given the current time step of the signal. During learning the two layers of the network specialized their rate constants differently to optimize the prediction of the signal (Fig. 8C). After successfully learning the task the network had developed a hierarchy of rate constants, with the first layer having a faster rate constant responding to quick changes in the signal and the second layer having a slower rate constant, responding to slower changes in the signal (Fig. 8B).

## Discussion

### Summary of results

We showed that rate-based RNN models can improve performance by including adaptive rate constants. In particular, we showed that a particular choice of the rate constants can in general increase performance with respect to the commonly used approximations where $\alpha _s$, $\alpha _r$ or both are set to 1. Furthermore, we showed that these rate constants can be learned efficiently via the BPTT algorithm. The learned rate constants recover the time scales underlying the original process, thus giving valuable insight in the temporal structure of the data. The activation function plays an important role in recovering the time scales. While a sigmoid activation function leads to recovery of both time scales, this is not the case for the ReLU activation function. The recovery of the time scales is independent of the number of hidden units in the network. Although a larger network is theoretically more flexible to compensate for suboptimal choices of rate constants, this does not change the ability of the network to recover the correct values. When the dynamics of a data set are governed by multiple time scales, the spread in rate constants is successfully recovered by a network where every unit can learn an individual rate constant. Since slower rate constants can lead to longer memory retention, we tested the ability of the networks to maintain memories over longer periods. The adaptive rate constants led to an increased memory capacity compared to the standard Elman approximation. Two layers of ARU units were stacked together to investigate whether a hierarchy of rate constants is learned from data composed of multiple time scales. Indeed we find that the first layer of the network learned to respond at a fast time scale, while the second layer learned to respond at a slow time scale, opening avenues to investigate such hierarchies of time scales using RNNs.

### Relation to previous work

These results build on previous attempts to enhance RNNs by exploiting the time scale of the dynamics of the data. Adaptive rate constants have been proposed in continuous neural networks as a way to increase the expressiveness of an RNN^28^. In this work a purely theoretical derivation was provided for learning rate constants and time delays of the continuous neural network units. Other work showed that equipping neurons in a network with different fixed rate constants, could be beneficial on tasks having both long-term and short-term temporal relations^37^. In this setting, however, rate constants could not be effectively estimated through training. Similarly, different fixed rate constants improved performance for recurrent spiking neural networks, approaching the performance of long short-term memory (LSTM) units^38^. More recent work has shown that, by stacking multiple RNN layers with progressively slower fixed rate constants, better performance was achieved on predicting motor patterns^39^. It has also been shown that adjusting rate constants by numerical integration outperformed the adjustment of time delays in the context of a chaotic process^29^. However, the computational cost of numerical integration hampered the extension to large-scale networks and real world problems. More recent work has attempted to create modules in an RNN, each with its own fixed temporal preference, to improve learning dynamical data with different temporal dependencies^40^. However, these time scales are fixed by the architecture’s connections and can not be optimized during training. Here we implemented the learning of rate constants using BPTT, with the benefit of easily extending the implementation of such time scales to more complex network architectures.

Studies into the role of time scales in the brain have revealed a temporal hierarchy, much like the spatial hierarchies found in the visual cortex^7^. Similar to the spatial hierarchy exhibited by the receptive fields of neurons in the visual cortex, there is also a temporal hierarchy of neurons responding to fast stimulus changes in early visual cortex, while neurons in higher visual cortex respond to slower changes. The possibility of developing a hierarchical temporal structure has also been suggested in the context of cell assemblies; generic densely interconnected groups of neurons that serve as representations of static or dynamic events of different duration^13,14^. Such assemblies can be combined sequentially to represent events of complex temporal structure^41,42^.

A tempting explanation for the emergence of such a hierarchy is that the hierarchical causal structure of the outside world shapes the representations of the brain^8,15–17^. Neural networks have been used to study the emergence of a hierarchy of time scales in producing motor patterns^39^, showing a functional role of higher layers with slow dynamics composing different motor primitives together in the lower layers with fast dynamics. However, such hierarchies were partially imposed by fixing the time scales of subsequent layers to progressively slower time scales. Here we show that when time scales are learned, such a hierarchy of time scales emerges automatically from the data, without the need for imposing any architectural constraints. These results provide promising avenues for investigating the emergence and functioning of a hierarchy of time scales in the brain.

### Benefits of learning rate constants

Learning rate constants can be useful when modeling neuroscientific data. To gain deeper insight in the functional role of the hierarchy of time scales in the visual cortex, neural networks with emerging hierarchies of time scales may be used as encoding models, similar to how spatial hierarchies were successfully modelled by feedforward convolutional networks^43^. By training RNNs with adaptive time scales to predict neural responses we can combine knowledge about the temporal structure of the cortex with insight in the actual information that is processed by a neural population. Questions regarding what properties of the data lead to the emergence of a hierarchy of time scales, and how task requirements can influence the time scales which certain cortical areas are responsive to can be investigated through such models and validated against experimental data.

Another example stems from the motor control literature. The motor patterns formed by the brain are thought to be composed of smaller motor ‘primitives’^25^. These primitives can be combined in a temporal sequence by a hierarchical process, where a slower process composes these primitives in meaningful motor patterns. Again, our approach could gain insight in which parts of the motor cortex represent these slower processes and which parts represent the faster primitives processes. Our approach can help us gain valuable insight in the functional relevance of the many dynamical processes in the brain.

Besides modeling neuroscientific data, learning rate constants can also be useful in machine learning applications. Building intelligent models to learn complex dynamical tasks, could benefit from artificial neurons that can learn and adapt to the time scales relevant for the problem at hand. Similar to the brain, artificial systems are often trained on natural data, where processes evolve over different time scales and certain information remains relevant over longer time scales while other information is only relevant for a very short period of time. An interesting application is the generation of natural movements for robots. It has been shown that using different time scales in a hierarchical model improves the learned motor patterns^25,39,44^. Similarly, the recognition of actions performed by humans from video data has been shown to benefit from such an temporal hierarchical structure^45,46^. However, setting such a hierarchy of time scales by hand is cumbersome and does not guarantee optimal results. Including the learning of these time scales in the optimization of the network ensures automatic optimization and could lead to the automatic emergence of models with relevant hierarchical time scales.

### Future work

Our approach enables researchers to build more expressive neural network models, and at the same time recover relevant temporal information from the data. Building towards large-scale models to predict brain responses over a large number of areas will gain us valuable insight in the dynamics of brain processes. Extensions towards learning time delays between brain areas or learning rate constants that can be modulated by the input are promising future steps. Keeping such complex models well-behaved during optimization, without falling prey to local optima, is an important challenge for future work. On the other hand there is the challenge of keeping networks interpretable by letting the network learn relevant biological parameters (such as rate constants or time delays). Further work is needed to investigate how well such parameters can be recovered in large and complex networks where such complex dynamics might emerge that learning the correct parameters can be circumvented^47^.

## Conclusions

We found that making standard RNN models more biologically plausible by introducing learnable rate constants improves performance of the model and increases its memory capacity, while at the same time enabling us to recover the time scales of the underlying processes from the data. Gaining explicit knowledge about the time scales at which processes unfold can improve our understanding of hierarchical temporal dynamics in the brain. At the same time, facilitate explicit time scales the creation of more expressive and interpretable machine learning models, which shows that embracing principles of neural computation can help us to develop more powerful AI systems^48^.

## Supplementary information

Supplementary Information.
