# Supplementary material for: Adaptive time scales in recurrent neural networks
Source: Sci Rep. 2020 Jul 9;10:11360. doi: 10.1038/s41598-020-68169-x (PMC7347927; doi:10.1038/s41598-020-68169-x)
Supplement: Supplementary file 1 — Supplementary Information. [file 41598_2020_68169_MOESM1_ESM.pdf]

Adaptive time scales in recurrent neural networks

Silvan C. Quax[1,*,+], Michele D'Asaro[1,+], and Marcel A. J. van Gerven[1]

[1]Donders Institute for Brain, Cognition and Behaviour, Radboud University, Nijmegen, The Netherlands
[*]corresponding author: s.quax@donders.ru.nl
[+]these authors contributed equally to this work

Supporting information

S1 Appendix Derivative of synaptic current.

The derivative of the synaptic current can be calculated using the Leibniz integral rule. Starting from Eq 6 and using the exponential kernel (Eq 3) we obtain

$$\begin{aligned}\frac{dI_n}{dt} &= \sum_k w_{nk}\int_{-\infty}^{t} d\tau \frac{e^{-\frac{(t-\tau)}{\tau_s}}}{\tau_s} r_k(\tau) \\ &= \sum_k w_{nk}\int_{-\infty}^{t} d\tau \frac{d}{dt}\frac{e^{-\frac{(t-\tau)}{\tau_s}}}{\tau_s} r_k(\tau) \;+\; \sum_k w_{nk}\frac{e^{-\frac{(t-t)}{\tau_s}}}{\tau_s} r_k(t)\cdot\frac{d}{dt}t \\ &= -\frac{1}{\tau_s}\sum_k w_{nk}\int_{-\infty}^{t} d\tau \frac{e^{-\frac{(t-\tau)}{\tau_s}}}{\tau_s} r_k(\tau) \;+\; \frac{1}{\tau_s}\sum_k w_{nk} r_k(t)\ ,\end{aligned} \tag{1}$$

S2 Appendix Linear activation function and symmetric time constants.

To understand why ReLU activation functions do not recover the time parameters well it could be informative to consider what happens when we make a linear approximation of the activation function. We start again with Eqs 8 and 9:

$$\tau_s\frac{dI_n}{dt} = -I_n + \mathbf{w}_n^\top\mathbf{r} + \mathbf{u}_n^\top\mathbf{x} \tag{2}$$

$$\tau_r\frac{dr_n}{dt} = -r_n + f(I_n)\ . \tag{3}$$

We use a linear activation function, that is $f(I) = I$. Rewriting Eq 23

$$I_n = \tau_r\frac{dr_n}{dt} + r_n \tag{4}$$

and computing the time derivative

$$\frac{dI_n}{dt} = \tau_r\frac{d^2r_n}{dt^2} + \frac{dr_n}{dt}\ , \tag{5}$$

it is possible to substitute expressions for I_n and $\frac{dI_n}{dt}$ into Eq 22 to get

$$\tau_s\left(\tau_r\frac{d^2r_n}{dt^2} + \frac{dr_n}{dt}\right) = -\left(\tau_r\frac{dr_n}{dt} + r_n\right) + \mathbf{w}_n^\top\mathbf{r} + \mathbf{u}_n^\top\mathbf{x} \tag{6}$$

$$(\tau_s\tau_r)\frac{d^2r_n}{dt^2} + (\tau_s+\tau_r)\frac{dr_n}{dt} = -r_n + \mathbf{w}_n^\top\mathbf{r} + \mathbf{u}_n^\top\mathbf{x}\ , \tag{7}$$

which is a single second-order differential equation, describing the firing rate dynamics only in terms of firing rates. We have two new time constants that govern the dynamics, $\tau_1 = (\tau_s + \tau_r)$ and $\tau_2 = (\tau_s\tau_r)$, which are invariant under exchange of τ_s, τ_r, and therefore symmetric. This explains why the time constants are not uniquely recoverable for ReLU units, but instead are exchangeable for one another.
